# In-vivo right ventricular myocardial perfusion assessment using BOLD and first-pass cardiac magnetic resonance

**DOI:** 10.1186/1532-429X-13-S1-P226

**Published:** 2011-02-02

**Authors:** David S Fieno, Steven M Shea, Yongzhong Li, Debiao Li

**Affiliations:** 1 Heart South Cardiovascular, Alabaster, AL, USA; 2 Northwestern University, Chicago, IL, USA; 3 Cedars-Sinai Medical Center, Los Angeles, CA, USA

## Introduction

Evaluations of right ventricle (RV) parameters are feasible using echocardiographic, nuclear, computed tomography and cardiovascular magnetic resonance (CMR) techniques but perfusion of the RV is not routinely assessed. Knowledge of blood flow to myocardium of the RV might be a useful parameter in the assessment of disease states. Such a measurement might yield important information about congenital, ischemic, and acquired conditions of the right heart.

## Purpose

We sought to determine whether blood oxygen level dependent (BOLD) and first-pass (FP) CMR during adenosine are useful techniques to measure perfusion in myocardium of the RV.

## Methods

**Figure 1:**
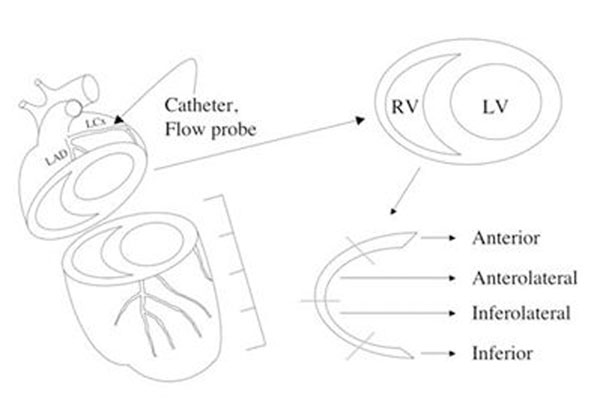
Experimental model showing coronary catheter, sectioning, and right ventricular segmentation:

Dogs (n=7) were prepared with a selective coronary artery catheter (Figure 1). Canines have left dominant circulation and the left circumflex supplies blood flow to the inferior RV myocardium. Thus, selective circumflex adenosine infusion in this model causes vasodilation of the inferior RV myocardial segment while blood from from the right coronary maintains normal blood flow in other areas. Animals were imaged on a 1.5-Tesla CMR scanner by BOLD and FP CMR before and after intracoronary adenosine (0.30 mg/min). Fluorescent microspheres were injected at rest and during adenosine stress through a left atrial catheter, implanted at the time of surgery. Image intensities of vasodilated and remote RV myocardium were measured. CMR perfusion indices were compared to relative perfusion of the RV based on fluorescent microsphere measurements.

## Results

**Figure 2:**
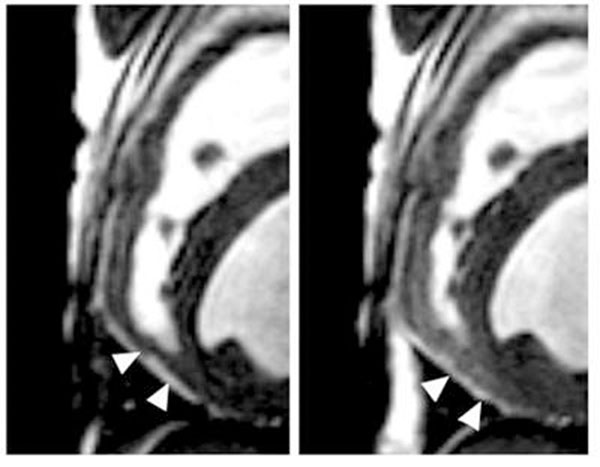
Example BOLD images of the RV (baseline shown at left, during adenosine shown at right) revealing increased image intensity (arrowheads) in the inferior segment during vasodilation:

**Figure 3:**
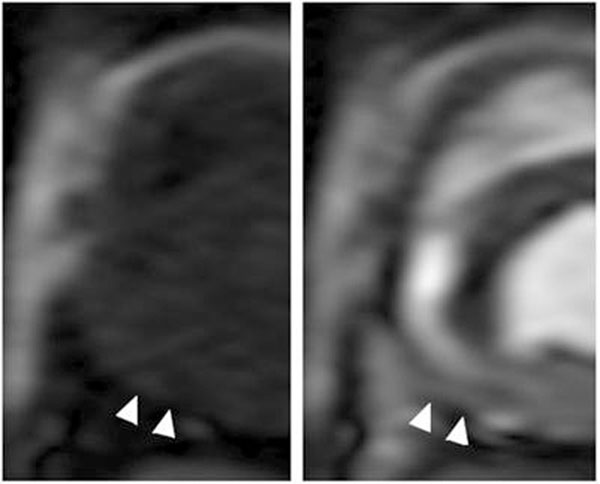
Example FP images of the RV (precontrast at left, during gadolinium at right) revealing increased image intensity (arrowheads) in the inferior segment during pharmacologic vasodilation.

Changes in RV myocardial perfusion during adenosine were apparent in BOLD and FP CMR images (Figures 2, 3). Stress-rest CMR perfusion indices of the RV correlated with that determined by microspheres (y=0.11x + 0.96, R=0.77, and y=0.91x + 0.44, R=0.81, for BOLD and FP-CMR, respectively).

## Conclusions

Data of the present study suggest that BOLD and FP CMR are useful techniques to assess of perfusion in the RV. To our knowledge, this is the first report of CMR to determine RV perfusion. In this animal model, BOLD and FP CMR during adenosine stress are non-invasive methods to assess RV myocardial perfusion.

